# Oral contraceptive use and premenstrual syndrome among sexually active female university students in Cameroon

**DOI:** 10.11604/pamj.2020.36.333.25078

**Published:** 2020-08-24

**Authors:** Derick Akompab Akoku, Thomas Achombwom Vukugah, Mbah Abena Tihnje, Idris Bigweh Nzubepie

**Affiliations:** 1 Health Alliance International, Abidjan, Côte d'Ivoire,; 2 Department of Global Health, University of Washington, Seattle, WA, USA,; 3 Elizabeth Glaser Pediatric AIDS Foundation, Yaounde, Cameroon,; 4 ICAP, Columbia University, Cameroon Office, Yaounde, Cameroon,; 5 Center for Global Health Practice and Impact-HIV Project, Georgetown University, Yaounde, Cameroon

**Keywords:** Oral contraceptive pills, premenstrual symptoms, menstruation

## Abstract

**Introduction:**

premenstrual syndrome (PMS) is a prevalent health problem affecting women of reproductive age and some young women use oral contraceptive pills (OCPs) to prevent unwanted pregnancy. However, the association between OCP use and the experience of symptoms of PMS has not been studied in Cameroon. We examined the association between the use of OCPs and PMS among female university students in Cameroon.

**Methods:**

we used data extracted from a larger study on sexual and reproductive health that was conducted between July and August 2018 among female university students at the University of Yaoundé 1, Cameroon. A pre-tested, validated and paper-based self-administered questionnaire was used to collect data. We extracted data (demographic and health characteristics, contraceptive use and experience of PMS) for the 424 sexually active students who participated in the larger study. We used Poisson regression analysis to examine the association between OCP use and PMS and conducted stratified analysis to determine effect modification. The level of statistical significance was set at p≤0.05.

**Results:**

the median age of the 424 sexually active female university students was 23 years (IQR=21-25). About 77.8% of participants self-reported to have experienced symptoms of PMS prior to their last menstrual period. The most commonly reported symptoms of PMS were breast tenderness (55.7%), acne/pimples (39.2%) and abdominal pain (31.1%). After adjusting for confounders in multivariate analysis, we found a statistically significant relationship between the use of OCPs and experience of symptoms of PMS. Current OCP users had a slightly increased risk (PR=1.21; 95%CI, 1.09-1.32, p<0.001) of developing symptoms of PMS compared to non-pill users. We found that age modified the effect of this association. Among older students (≥25 years), the direction of the effect was strongly positive and statistically significant (APR=1.32; 95%CI, 1.12-1.56, p=0.001).

**Conclusion:**

the proportion of female university students who reported to have experienced symptoms of PMS prior to their last menstrual period was high. The use of OCPs was positively associated with the risk of developing symptoms of PMS and this relationship was modified by age. Further studies in Cameroon and other sub-Saharan African countries are required to understand this relationship.

## Introduction

Across the world, premenstrual syndrome (PMS) is a common health problem affecting women of reproductive age [1,2]. PMS has been defined as a complex of emotional, physical and behavioral symptoms that start at the last week of a woman´s reproductive cycle and disappears at the onset of menstruation [1,3]. An estimated 75% of women who menstruate report PMS and the rate is higher among young women [4]. The extreme and predominantly psychological form of PMS is called premenstrual dysphoric disorder (PMDD) which affects about 3-8% of women with PMS [5]. A systematic review and meta-analysis estimated that the prevalence of PMS was 47.8% around the world [3]. Other studies in Asia have reported prevalence rates which are higher than 59% [6,7]. Studies conducted in sub-Saharan Africa (SSA) have reported that PMS are quite common among female university students [8-11]. For example, a study conducted in Ethiopia among 300 female university students found that the prevalence of PMS was 67%, but only 9.7% of those who experienced PMS consulted a health practitioner to seek care [6]. Although PMS is not life threatening, it has been reported to decrease the quality of life of women and impairs social relationships with friends and family [6,8,12]. PMS has also been found to negatively impact school functions (e.g. missed classes and missed exams). In a study conducted in Eritrea, Azaria and colleagues found that among the students who were diagnosed with PMS, 78.6% reported that their concentration in class was affected, 90.5% indicated that they stopped studying, while 45% reported missing classes [8].

In Cameroon and other countries in Africa, female university students use contraceptives to prevent unwanted pregnancy and oral contraceptives pills (OCPs) are among the most widely used hormonal contraceptives [8,13]. During the natural menstrual cycle, female gonadal hormones (estrogen and progesterone) change in a regular temporal pattern which facilitates conception. The role of OCPs containing a combination of an estrogen and progestogen is to suppress ovulation by modifying the natural fluctuation of these hormones [14-16] thereby preventing pregnancy. Although the etiology of PMS remains unclear, it has been suggested that genetic, environmental, and psychological factors contribute to increased sensitivity to normal hormonal changes and neurotransmitter abnormalities which lead to PMS [15,17]. The relationship between OCPs and the development of symptoms of PMS (e.g. depression, anxiety and mood disorders e.t.c.) are inconclusive. In developed countries, studies have examined the relationship between OCP use and the development of symptoms of PMS [18-20]. One study established a positive association between OCP use and general or symptoms of PMS [19]. However, another study in the United States suggested that users of hormonal contraception reported lesser symptoms of PMS than non-users [21].

A previous study did not find any association between OCPs use and the development symptoms of PMS [22]. Although studies have estimated the prevalence of PMS among female university students in many settings across SSA [8-11], none of these studies examined the effects of exogenous hormones (e.g. oral contraceptive pills) on the development of symptoms of PMS. A review of the academic literature suggests that to-date, there is no published study on PMS from Cameroon, although the condition affects the health and wellbeing of many women in the country. As a result, we do not know estimates of the proportion of women who experience PMS. Furthermore, little is known about the potential associations between OCP use and symptoms of PMS despite the fact that many young women in Cameroon use OCPs to prevent unwanted pregnancy. The present paper addresses the limited evidence of the relationship between OCP use and PMS in Cameroon. The purpose of this study was to estimate the prevalence of PMS and determine whether the use of OCP was positively associated with PMS among female university students in Cameroon. The current paper is a substudy of a larger study that examined the sexual and reproductive health of female university students. Our findings may contribute to inform programs and policies on reproductive health education and contraceptive use among young women in the country.

## Methods

This paper is based on a sub-sample of data from a larger study that examined the sexual and reproductive health of female university students in Cameroon. In the following section, we describe the detailed methods of the larger study and explain how we extracted data for the current paper/analysis. The larger study was a cross-sectional study that was conducted at the University of Yaounde I, Cameroon. The university was formed in 1993 following university reforms that split the country's oldest university, the University of Yaounde, into two separate entities: the University of Yaounde I and the University of Yaounde II. The University of Yaounde 1 offers both undergraduate and graduate programs to over 55,000 students. It has four faculties: faculty of science; faculty of medicine and biomedical sciences; faculty of education; and the faculty of arts, letters and human sciences [23].

**Study population and sampling:** the larger study was conducted among female students at the University of Yaounde 1, Cameroon. The sample size was estimated using the single population proportion formula for cross-sectional studies. The minimum sample size was derived from the formula:

$$n=\frac{\left[z^{2}xp\left(1-p\right)\right]}{m^{2}}$$

where n=the minimum required sample size for the study; z=the standard deviation for a 2-tailed test at 95% confidence level (1.96); p(53%)=the estimated study parameter; m=the margin of error (4%) which gives a sample size of 600 participants. We assumed a 13% non-response rate; as a result, the minimum sample size (n) estimated was 690 participants. This was the minimum required sampling units which would be required to build sound statistical conclusion and inferences. Nevertheless, we increased the final sample size in order to boost the statistical power of the study[24]. A convenience sampling technique was used to recruit participants for the larger study. However, efforts were made to collect data from a representative sample of students taking into account relevant background characteristics (e.g. age, level of study and other relevant demographic characteristics e.t.c.). For the larger study, participants were considered eligible if: they were a female student at the university; able to read and write in French or English and; willing to provide written informed consent in French or English. We excluded medical students and students in health-related disciplines because they are more likely to practice positive healthy behaviors and their responses could bias the study results.

**Data collection:** the study team recruited and trained research assistants on the objectives of the study, maintaining confidentiality, participants´ right and informed consent. A draft questionnaire was developed in English after reviewing the literature. It consisted of both closed and open-ended questions. The draft questionnaire was shared with 10 experts/professionals with experience working in the area of sexual and reproductive health for face and content validity. Feedback and inputs from these experts guided the revision of the questionnaire. The questionnaire was pre-tested among 35 female university students attending a private university in Yaounde. After the pre-test, all difficult or ambiguous questions were deleted or reworded and relevant modifications were made. The final validated questionnaire was translated into French and later back translated into English and any discrepancies were reconciled. At the university campus, research assistants approached and screened students for eligibility. Eligible participants who provided written informed consent were provided with the validated paper-based questionnaire (Cronbach alpha=0.81). Participants who preferred to complete the questionnaire in French were given the French version while those who preferred to complete in English were given the English version of the questionnaire. Prior to data collection, participants were explained the purpose of the study, their rights to participate as well as to opt-out from the study at any time. Participation was entirely voluntary and students were assured of the confidentiality of their responses, their right to decline or withdraw from the study at any time. No names, addresses or personal information were collected.

**Ethical considerations:** the study protocol for the larger study was reviewed and approved by the Cameroon National Research Ethical Committee for Human Health (No: 2018/07/1084/CE/CNERSH/SP). In addition, administrative authorization was obtained from the rector of the University of Yaounde 1 to collect data among female students at the campus. The anticipated benefits or risks were clearly explained to the participants and they provided written informed consent to indicate their willingness to take part in the study. Voluntary participation, confidentiality and anonymity were strictly observed.

**Figure 1:**
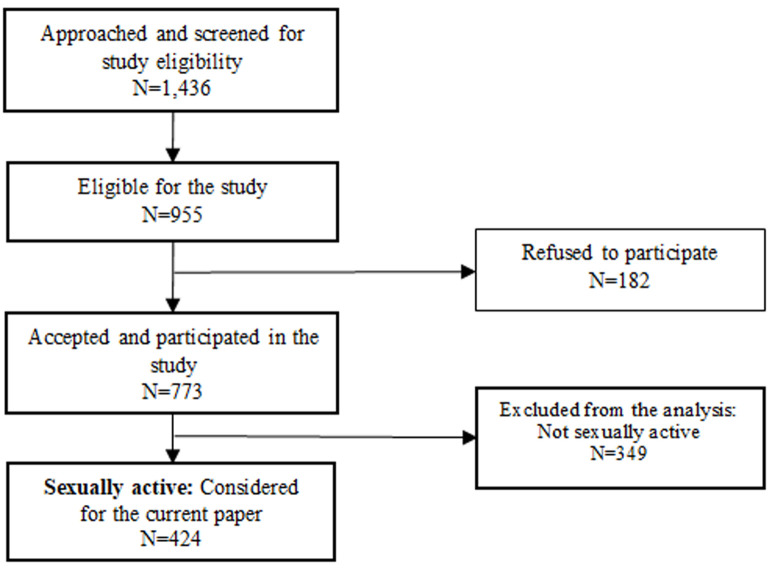
study flow diagram and data extraction for current paper

**Data extraction for the current paper and variables of interest:** Figure 1 shows the flow diagram of the larger study and the subsample of data extracted for the current paper. Of the 993 students who met study eligibility, 773 accepted to participate in the study (response rate 81%). The study team extracted data for the 424 (54.9%) sexually active female university students who participated, while those who were not sexually active (45.1%) were excluded in the current analysis. To enable us answer the research questions we also extracted relevant variables (outcome, exposure and covariates) from the database as outlined below:

**Outcome variable:** the primary outcome variable was: “self-reported experience of PMS prior to last menstrual period”. During the larger study, participants were provided a definition of PMS: “women usually experience some physical and emotional symptoms about two weeks prior to the onset of menstruation”. They were later asked: *did you experience any symptoms or observed physical changes before the start of your last menstrual period?* The response option was “yes/no”. Those who responded “yes” were presented with a list of common symptoms of PMS and asked to identify one or more types of PMS which they experienced prior to their last menstrual period.

**Exposure variable:** the exposure variable in our analysis was OCP use. This was measured during the larger study by asking participants: “in the past 3 months, have you been using oral contraceptive pills to prevent unwanted pregnancy? (yes/no)”. Participants who indicated “yes” were considered as OCP users while those who indicated “no” were considered as non-users.

**Covariates:** the following variables were extracted from the database and investigated as potential confounders and/or effect modifiers: age, marital status, year of study (undergraduate, postgraduate), experience of financial hardship in the past 3 months (yes/no), age at first sex, age at first menarche (early menarche in this study was defined as first menstruation at 12 years or younger and late menarche as 13 years or older) (Figure 1).

**Statistical analysis:** the extracted data for the 424 sexually active female university students were analysed using STATA version 14.0 (Stata Corp, College Station, Texas, USA). Continuous variables were summarized by median and inter-quartile range (IQR) while categorical variables were summarized using frequencies and percentages. We performed multivariate models to examine the relationship between our exposure variable (OCP use) and the outcome variable (self-reported PMS). We estimated the Prevalence Ratio (PR) via a modified Poisson regression model using a generalized linear model with robust variance estimation. The robust variance estimation is usually used to avoid overestimating standard errors of parameter estimates (Yelland *et al*., 2011). We used prevalence ratio because odds ratio may not be a good measure of association given its potential to overestimate the effect as the primary outcome of our study was common (Tamhane *et al*., 2016).We examined the effect of each of the confounders on the relationship between OCP use and PMS. Finally, we fitted the complete model adjusting for age, marital status, level of study, experience of financial hardship in the past 3 months, age at first sex and age at first menarche. We also examined our data for effect modification to determine whether age modified the relationship between the exposure and the primary outcome. We created a binary variable for age: i.e. younger age (<25 years) and older age (≥25 years) in the stratified analysis. Prevalence ratio (PR) and 95% Confidence Interval (CI) were calculated to estimate the strength of the relationship. All statistical tests performed were two-tailed and a p≤0.05 was considered statistically significant.

## Results

**Participants´ characteristics:** Table 1 shows the characteristics of the sexually active female university students who participated in the study. The median age of participants was 23 years (IQR=21-25) and 65.8% were less than 25 years old. About 83.2% of the students were single and 79.0% experienced financial hardship in the past 3 months. The median age at first sex was 18 years (IQR=17-20) while the median age of first menses was 13 years (IQR=12-14). About 25.2% of the students reported to have used an OCP in the past 3 months, while 77.8% reported to have experienced one or more symptoms of PMS prior to their last menstrual period.

**Table 1:** participants' characteristics (n=424)

Characteristics	Number (N)	Frequency (%)
**Used oral contraceptive pills in past 3 months**	****	****
No	317	74.8
Yes	107	25.2
**Experienced PMS prior to last menstrual period**	****	****
No	94	22.2
Yes	330	77.8
**Age group (years)**	****	****
<25	279	65.8
25+	145	34.2
**Marital status**	****	****
Single	353	83.2
Married	71	16.8
**Experienced financial hardship in past 3 months**	****	****
No	89	21.0
Yes	335	79.0
**Level of studies**	****	****
Undergraduate	319	75.2
Postgraduate	105	24.8
**Age at sexual debut**	****	****
<15	21	4.9
15-20	351	82.8
21+	52	12.3
**Age at first menses**	****	****
6-12yrs	189	44.6
13-18yrs	235	55.4

**Figure 2:**
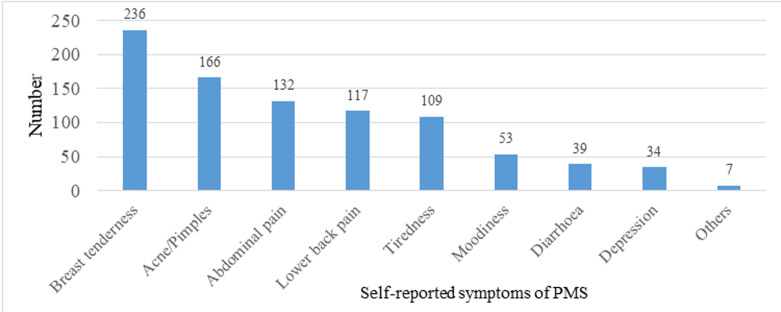
self-reported symptoms of PMS prior to last menstrual period (n=424)

**Common symptoms of PMS experienced:** Figure 2 shows the common forms of self-reported PMS indicated by participants. The most common symptoms experienced were breast tenderness 55.7% (236/424), acne/pimples 39.2% (166/424) and abdominal pain 31.1% (132/424). Diarrhea and depression were the least commonly reported symptoms of PMS. Other symptoms 1.7% (7/424) experienced included feeling hopeless, headache, insomnia, food cravings and nausea.

**Oral contraceptive use and PMS:** Table 2 shows the association between OCP use and PMS. In unadjusted models, we found that the prevalence of PMS was higher among OCP users than non-users (PR= 1.21; 95%CI, 1.11-1.33, p<0.001). In multivariate models, we found evidence of a strong association between using OCP and PMS. The prevalence of PMS was significantly higher among OCP users (APR=1.21; 95%CI, 1.09-1.32, p<0.001) compared to their peers who did not use OCP. With regards to the relative impact of each confounding on the association, experience of financial hardship in the past 3 months had the largest impact on effect size on the relationship between OCP use and PMS. Table 3 shows stratified analysis by age for the relationship between OCP use and experience of PMS (i.e. to determine if the effect of OCP use on risk of developing PMS was different among younger and older participants). We found evidence of effect modification and the direction of effects were consistent in both substrata and was statistically significant. The multivariate adjusted prevalence ratio of experiencing PMS among OCP users compared with non-users was lowest for younger aged (<25 years) students (APR=1.16; 95% CI, 1.03-1.30) and increased steadily for older (≥25 years) students (APR=1.32; 95%CI, 1.12-1.56).

**Table 2:** unadjusted and adjusted relationships between OCP use and PMS

Measure	PR (95%CI)	p-value
Unadjusted association	1.21 (1.10-1.33)	p<0.001
Adjusted for age1	1.21 (1.11-1.33)	p<0.001
Adjusted for marital status	1.22 (1.11-1.33)	p<0.001
Adjusted for level of studies	1.22 (1.11-1.33)	P<0.001
Adjusted for financial hardship	1.20 (1.09-1.32)	p<0.001
Adjusted for age at first sexual debut	1.22 (1.11-1.33)	P<0.001
Adjusted for age at first menarche	1.21 (1.10-1.32)	p<0.001
Adjusted for all2	1.21 (1.09-1.32)	P<0.001

1 Age was considered as a continuous variable; 2 Adjusted for age, marital status, level of studies, financial hardship in the past 3 months, age at first sexual debut and age at first menarche

**Table 3:** association between OCP use and PMS, stratified by age

Measure	Younger age (<25 yrs)		Older age (≥25 yrs)	
****	**PR (95%CI)**	**p-value**	**PR (95%CI)**	**p-value**
Unadjusted association	1.16(1.04-1.31)	0.007	1.31(1.12-1.54)	0.001
Adjusted for marital status	1.18(1.05-1.32)	0.005	1.31(1.12-1.54)	0.001
Level of studies	1.16(1.04-1.30)	0.007	1.32(1.12-1.55)	0.001
Adjusted for financial hardship	1.15(1.03-1.29)	0.014	1.30(1.11-1.53)	0.001
Adjusted for age at first sexual debut	1.18(1.05-1.32)	0.004	1.31(1.12-1.53)	0.001
Adjusted for age at first menarche	1.16(1.04-1.30)	0.008	1.32(1.12-1.56)	<0.001
Adjusted for all	1.16(1.03-1.31)	0.011	1.32(1.12-1.56)	0.001

## Discussion

The purpose of our study was to estimate the prevalence of PMS and examine the relationship between OCP use and PMS among female university students in Cameroon. To our knowledge, this is the first study in Cameroon to investigate the association between OCP use and PMS among young women. Overall, we found that PMS was prevalent as 77.8% of the participants reported to have experienced PMS before their last menstrual period. We also found a positive and significant relationship between OCP use and PMS. The high rate of PMS found in this study is consistent with previous studies which have also reported a high prevalence of PMS among young women in Ethiopia [6,11]. Although the prevalence of PMS in this study was high, previous studies have reported lower rates of PMS among young women. For example, a study among 200 medical students in Eritrea found that the prevalence of PMS was 17.5% [8]. Similar studies conducted in India among 489 college girls demonstrated that 18.4% reported PMS [25]. The differences in the prevalence of PMS reported in research studies have been attributed to differences in the sample size, study population, the geographical context, the tool used to diagnose PMS and the truthfulness with which study participants report their symptoms [2,26]. Our study found an association between the use of OCPs and experience of symptoms of PMS. In unadjusted models, female students who were current users of OCPs were more likely to have reported symptoms of PMS compared to their peers who did not use OCPs. Nonetheless, the crude associations observed could have been due to confounding factors. However, after adjusting for confounding factors there was still a strong relationship between OCP use and PMS. The association between OCP use and PMS may be attributed to some of the mild side effects associated with OCP use and some of the symptoms of PMS are among the side effects associated with OCP use [27].

Nevertheless, our finding of the association between OCP use and PMS contrast previously published studies in the academic literature. For instance, a study conducted among 2,115 female university students in Lebanon did not find any association between OCP use and PMS [26]. A similar study among 490 female university students in Brazil, did not find any association between OCP use and PMS [28]. Our findings add to the existing conflicting evidence regarding a potential association between OCP use and development of symptoms of PMS which underscores the need for similar studies in Cameroon and other African countries. In Western societies, many studies have examined the association between OCPs and symptoms of PMS (e.g. depression, mood changes e.t.c.). Some studies have reported that women taking OCP are more likely to be depressed than non-pill users[29,30]. If these studies could establish an association between OCPs use and depression (which is a somatic symptom of PMS), then there is a likelihood that the use of OCPs is associated with PMS.

However, it is important to keep in mind that although these studies established an association between the use of OCPs and depression, it is unclear whether depressive symptoms were assessed during or after the luteal phase of the menstrual cycle to distinguish whether it was premenstrual syndrome-related depression or generalized depression [31]. In fact, women suffering from PMS-related depression and PMDD have reported dramatic relief from their symptoms once their menstrual flow begins [17]. Although our study established a relationship between OCP use and the experience of PMS, it has been reported that OCPs (used to suppress ovulation and regulate the menstrual cycle) could be used in the treatment of PMS, although the results from these studies have been mixed with low quality of evidence[16,32]. For example, our study found that acne/pimples was among the most commonly reported symptoms of PMS. Paradoxically, OCPs (containing estrogen and progestin) have previously been prescribed to women for the treatment of acne [33]. Our findings therefore add to the conflicting evidence on the potential benefit of OCPs in the treatment of PMS [16] which underscores the need for more clinical studies.

Our findings should be interpreted bearing in mind the following limitations. Firstly, data for the analysis was derived from a cross-sectional study. As a result, no definite conclusions on the causal (cause-effect) relationship can be established, as association does not imply causation. Secondly, this was an observational study and not a randomized controlled trial, therefore other significant confounding factors which were not examined in this study may have influenced the relationship. Thirdly, PMS was assessed using a self-administered questionnaire rather than a standardized diagnostic tool for assessing PMS. The use of self-administered questionnaires may have led to response bias with students either under or overestimating their experience of PMS. Fourthly, a convenience sample was used to recruit study participants which suggest that the sample may not accurately represent the entire female student population at the University of Yaounde 1, Cameroon. In spite of these limitations, our findings will generate interest and stimulate discussion on the potential association between OCP use and the risk of developing PMS which may prompt future research studies (e.g. randomized control trials) in Africa to clarify this relationship.

## Conclusion

Our study found that PMS was prevalent among female university students which underscores the need for interventions to help them properly manage these symptoms. There was a statistically significant association between OCP use and PMS. Given that OCP are among the most commonly used modern contraceptive method by female university students, health providers should counsel these students on the potential benefits and minor side effects of OCP use. Despite our findings, reproductive health programs should promote access to safe, affordable and convenient contraceptive methods to enable female university students prevent unwanted pregnancy.

### What is known about this topic

- Symptoms related to premenstrual syndrome are common among women of childbearing age;
- The prevalence of premenstrual syndrome varies from one geographic region to another and among population groups;
- Hormonal contraceptives (e.g. oral contraceptives) are used to prevent unwanted pregnancy among women.

### What this study adds

- Premenstrual syndrome is prevalent among female university students;
- Exist a non-causal relationship between the use of oral contraceptive pills and the risk of experiencing symptoms of prementrual syndrome;
- Identification of risk factors for PMS among young women is important to reduce the occurrence of this health condition.
